# Psychological Distress in Healthcare Workers between the First and Second COVID-19 Waves: The Role of Personality Traits, Attachment Style, and Metacognitive Functioning as Protective and Vulnerability Factors

**DOI:** 10.3390/ijerph182211843

**Published:** 2021-11-11

**Authors:** Loredana Cena, Matteo Rota, Stefano Calza, Jessica Janos, Alice Trainini, Alberto Stefana

**Affiliations:** 1 Observatory of Perinatal Clinical Psychology, Department of Clinical and Experimental Sciences, Section of Neuroscience, University of Brescia, Viale Europa 11, 25123 Brescia, Italy; jjanos@live.unc.edu (J.J.); alice.trainini@unibs.it (A.T.); alberto.stefana@gmail.com (A.S.); 2 Unit of Biostatistics, Biomathematics and Bioinformatics, Department of Molecular and Translational Medicine, University of Brescia, Viale Europa 11, 25123 Brescia, Italy; matteo.rota@unibs.it (M.R.); stefano.calza@unibs.it (S.C.); 3 Department of Psychology and Neuroscience, University of North Carolina at Chapel Hill, Chapel Hill, NC 27599, USA

**Keywords:** COVID-19, pandemic, psychological distress, burnout, healthcare workers, personality traits, attachment style, metacognitive functioning

## Abstract

The COVID-19 pandemic has impacted the mental health of healthcare workers (HCWs) since its outbreak, but little attention has been paid to person-level vulnerability and protective factors. This study aims to determine the prevalence of both general and pandemic-related psychological distress among HCWs between the first and second COVID-19 waves in Italy and analyze associations between psychological distress and personality traits, attachment style, and metacognitive functioning. Between June and October 2020, 235 Italian HCWs completed questionnaires concerning psychological stress, personality traits, attachment style, and metacognitive functioning; 26.5% of respondents presented with moderate to extremely severe levels of general psychological distress and 13.8% with moderate to extremely severe levels of pandemic-related psychological distress. After controlling for demographic and occupational variables, significant associations emerged among high emotional stability as a personality trait and both general (aOR: 0.58, 95% CI: 0.41–0.79) and pandemic-related psychological stress (aOR: 0.62, 95% CI: 0.41–0.90). Additionally, higher scores regarding one’s ability to understand others’ emotional states were associated with lower odds of developing psychological distress (aOR: 0.78, 95% CI: 0.63–0.93). Lastly, when comparing those with fearful attachment styles to those with secure attachments, the aOR for psychological distress was 4.73 (95% CI: 1.45–17.04). These results highlight the importance of conducting baseline assessments of HCWs’ person-level factors and providing regular screenings of psychological distress.

## 1. Introduction

During the ongoing global public health emergency, there has been a rapidly growing number of studies aimed at investigating the psychological impact of the COVID-19 pandemic, including vulnerabilities and protective factors in the general population [1,2,3] as well as in specific populations [4,5,6,7] and health care services [8]. Among these specific populations, healthcare workers (HCWs) experience much higher pressure than usual due to increased workload and risk of infection [9,10], in addition to the risk factors experienced by the general population [11,12]. As a result, HCWs face an increased risk of onset or worsening of mental health issues such as stress, anxiety, depression, rage, denial, somatization, and sleep disturbance [13,14] that may compromise the quality of their care delivery [15,16] as well as their psychological well-being [17]. Furthermore, it must be noted that working clinically with patients can evoke intense emotional reactions [18,19,20,21,22] that, if not managed properly, can affect the quality of HCWs’ work and lead to emotional biases in decision-making processes [23,24], which, in turn, may result in errors and adverse events [25,26]. Indeed, it has been found that physicians’ emotional responses can negatively influence medical safety [27,28,29]. Effective management of emotional responses occurs only when an individual is capable of tolerating and recognizing these responses and integrating them into a matrix of professional understanding [30,31]. Given that the pandemic may trigger the onset or worsening of psychological distress [12,32,33,34], there is a considerable need for research that enables a deeper understanding of risk and protective factors for the mental health problems of HCWs during the COVID-19 pandemic, which may help to inform support interventions for HCWs that will, in turn, have a positive impact on their patients.

Regarding vulnerability and protective factors of psychological distress, a growing number of studies have found that a person’s individual characteristics, such as personality traits, may account for some of the variances in people’s (including HCWs’) reactions to the pandemic [35,36,37,38], which is consistent with findings from pre-pandemic studies [39,40]. Although there is sufficient research on the relationship between personality traits and mental health, the relationship between personality traits and both attachment style and metacognitive functioning during the COVID-19 pandemic is still under-developed and usually focused on the general population rather than HCWs specifically.

### Aim of the Current Study

In the light of the above, this study focuses on the role of personality traits, attachment styles, and metacognitive functioning as vulnerability or protective factors for both general and pandemic-related psychological distress in HCWs. Given our general goal of examining personal vulnerability and protective factors in predicting psychological distress symptoms during the COVID-19 pandemic, we tested two models: one for general psychological distress and one for pandemic-related psychological distress. We also controlled for a series of demographic and occupational variables, including age, gender, professional role, and employment status. We hypothesized that: (1) levels of psychological distress symptoms among HCWs would be high, (2) emotional stability would be negatively associated with symptoms of both general and pandemic-related psychological distress, such that those with high emotional stability would have lower distress symptom ratings, (3) fearful attachment style would be positively correlated with higher levels of pandemic-related psychological distress, and (4) high metacognitive functioning would be negatively correlated with both general and pandemic-related psychological distress (we did not have specific hypotheses about the individual components of metacognition).

## 2. Materials and Methods

### 2.1. Study Design

The web-based survey used in this study was based on non-probability and a snowball sampling design. An invitation email with an electronic link to the survey was sent to the mailing lists of the main Italian perinatal healthcare professional associations and registers. Those healthcare workers who responded to the invitation were included in the study if they met the eligibility criteria: being at least 18 years old and having provided clinical care during the COVID-19 pandemic. Both the invitation email and the first page of the survey included an explanation of the study’s purpose, its voluntary nature, and the assurance of anonymity and confidentiality. Participants were informed that continuing beyond the first page of the questionnaire indicated informed consent. The questionnaire was administered via the LimeSurvey platform version 3.15.9+190214 (SoGoSurvey, Herndon, VA, USA) from June to October 2020. Data were internally validated to ensure that participants completed the questionnaire only once. The study was approved by the Ethics Committee of the ASST Spedali Civili Hospital (Brescia, Italy) on 24 June 2020 (ethical number: NP4221).

### 2.2. Survey Description

The questionnaire was designed by the members of the Observatory of Perinatal Clinical Psychology (University of Brescia: https://www.unibs.it/it/node/988, accessed on 13 September 2021) of the Department of Clinical and Experimental Sciences (University of Brescia, Brescia, Italy) and consisted of two parts: the first gathered basic socio-demographic and work data (see the variables reported in Table 1), whereas the second assessed mental health status through four validated self-rating scales.

The Italian version of the Depression, Anxiety, and Stress Scale-21 (DASS-21) [41,42] is a self-report 21-item scale to measure general psychological distress in both clinical and non-clinical samples. Items are scored on a spectrum ranging from 0 (“did not apply to me at all”) to 3 (“applied to me most of the time”). High scores indicate increased distress. For the purposes of this study, psychological distress, as measured by the DASS-21 scale, was dichotomized by grouping “normal and mild levels” and “moderate to extremely severe levels” using the cutoff <30 vs. ≥30 [43]. The internal consistency in the current study was Cronbach’s alpha = 0.94.

The Italian version of the Impact of Event Scale-Revised (IES-R) [44,45] is a 22-item self-report scale designed to measure trauma-related distress. Respondents were asked to indicate their degree of distress during the past seven days related to the COVID-19 pandemic. It is rated on a 0 (“not at all”) to 4 (“extremely”) Likert scale with respect to how distressing (rather than how frequent) each item has been. High scores indicate increased distress. The IES-R scales were dichotomized by grouping “normal and mild levels” and “moderate to extremely severe levels” using the cutoff <33 vs. ≥33 [46]. In our internal consistency analysis for the IES-R, we found a Cronbach’s alpha of 0.94.

The 10-item short version of the Big Five Inventory (BFI) [47,48] is a self-report measure of the Big Five dimensions of human personality, i.e., (1) agreeableness, (2) conscientiousness, (3) emotional stability (the inverse of neuroticism), (4) extraversion, and (5) openness. The inventory consists of 10 short-phrase items assessed on a 5-point Likert scale, ranging from 1 (“disagree strongly”) to 5 (“agree strongly”). The Italian version of the 10-item BFI showed an acceptable internal consistency (Spearman–Brown coefficients ranging 0.70–0.80) [48]. It must be noted that the Spearman–Brown coefficient performs better than Cronbach’s alpha in measuring the internal consistency of two-item subscales [49].

The Metacognitive Functions Screening Scale items (MFSS) [50] is a self-report measure assessing metacognitive functions. Metacognitive functioning can be defined as a set of abilities that allow a person to (a) attribute and recognize one’s own and others’ mental states (considering non-verbal communication and somatic states), (b) reflect and reason on one’s own and others’ cognitive and mental states, and (c) consciously and intentionally use information about one’s own mental state to regulate action plans in order to solve psychological and interpersonal problems or conflicts and manage personal suffering [51]. The MFSS’s items are scored on a spectrum ranging from 0 (“absolutely false”) to 3 (“absolutely true”). The measure comprises four subscales: (1) the ability to understand others’ emotional states (CRE), (2) the ability to understand causal relationships (CRC), (3) the ability to judge the distance between objects or the distance between oneself and an object (CDD), and (4) the ability to ponder situations and problems (CDP). In the present study, we found that Cronbach’s alphas for the four subscales were 0.82, 0.67, 0.57, and 0.69, respectively.

The Relationship Questionnaire (RQ) [52,53] is a 4-item self-report scale devised to measure four categories of adult attachment: (1) dismissive, (2) fearful, (3) preoccupied, and (4) secure. It is composed of four paragraphs describing each of the prototypical attachment orientations. This study’s respondents were asked to rate the extent to which each description resembled their general relationship style on a 7-point scale from 1 (“not at all like me”) to 7 (“very much like me”). Cronbach’s alpha values range from 0.32 for secure attachment to 0.79 for fearful attachment. The Italian translation of the RQ was developed following World Health Organization and Mapi Research Trust recommendations.

### 2.3. Statistical Analyses

Continuous variables are presented as mean ± standard deviation (SD) and range, while categorical variables are presented as frequencies and percentages. The associations between a-priori selected variables and moderate to extremely severe levels of psychological distress were assessed through univariate and multivariate logistic regression models. Results are presented in terms of odds ratios (ORs), 95% confidence intervals (CIs), and *p*-values. An OR greater than 1 indicates that moderate to extremely severe levels of psychological distress are more likely to occur within the considered explanatory variable. Conversely, an OR less than 1 indicates that moderate to extremely severe levels are less likely to occur.

Statistical analyses were processed through R version 4.0.2 (R Foundation for statistical computing, Vienna, Austria).

## 3. Results

### 3.1. Characteristics of the Sample

We received responses from 235 healthcare professionals, including midwives (30.2%), psychologists (24.7%), physicians (14.5%), and “other positions” (30.6%), which included nurses, nursery nurses, social-health assistants, physiotherapists, psychomotor therapists, and speech therapists. The majority of the participants (93.2%) were women, and the mean age was 44.4 years old (SD = 11.5). Most worked in community-based facilities (69.4%), while the remaining worked in hospitals (30.6%); 39.1% of participants had more than 16 years of work experience as a healthcare professional, while 30.6% had 6–15 years and 30.2% had less than 5 years. Most of the participants (74.5%) had permanent employment, while a minority worked as freelancers or had temporary employment contracts (25.5%). During the COVID-19 pandemic, less than half of the participants (40.3%) worked as usual, and the remaining two-thirds worked more than usual (29.2%) or less than usual (30.5%). Only a minority of responders worked in a COVID-19 unit (5.5%). Table 1 shows the details of the socio-demographic and work characteristics as well as the average and standard deviations of the investigated clinical variables (i.e., Big Five dimensions of human personality, attachment style, and metacognitive functions).

### 3.2. Severity and Scores of General and Pandemic-Related Psychological Distress

One hundred and ninety-six participants completed the Depression, Anxiety, and Stress Scale-21 (DASS-21) [42,54]. The mean score for general psychological distress was 44.7 (SD = 11.3), and 26.5% of HCWs reported symptoms above the cutoff for clinical significance. Regarding factors related to psychological distress, we found significant differences in symptomatology levels between people who had different attachment styles (*p* = 0.001), different levels of ability to understand others’ emotional states (*p* < 0.001) and to understand causal relationships (*p* < 0.001) as well as between different scores for two dimensions of the structure of personality, emotional stability (*p* < 0.001), and extraversion (*p* = 0.013).

Two hundred and twenty-four participants completed the Impact of Event Scale-Revised (IES-R) [44,45]. The mean score for trauma-related distress was 44.6 (SD = 11.5), and 13.8% of HCWs reported symptoms of distress above the cutoff for clinical significance. We found significant differences in symptomatology levels among people with different professional roles (*p* = 0.008), those who had permanent employment versus those who did not (*p* = 0.038), and those who continued to work during the pandemic versus those who did not (*p* = 0.049). Different levels of trauma-related distress were also found between different levels of ability to understand others’ emotional states (*p* = 0.005) and to understand causal relationships (*p* = 0.034) as well as between different scores for two dimensions of the structure of personality, emotional stability (*p* = 0.019), and openness (*p* = 0.037).

Table 2 shows the statistically significant socio-demographic, occupational-related, and person-level differences and the prevalence of both general and pandemic-related psychological distress. Table S1 reports all the variables considered in the analysis.

### 3.3. Independent Vulnerability and Protective Factors

The univariate regression analysis (Table 3) revealed that having higher scores in the abilities to understand others’ emotional states (CRE) and understand causal relationships (CRC) was associated with lower symptoms of both psychological distress (OR: 0.77, 95% CI: 0.68–0.86; OR: 0.81, 95% CI: 0.73–0.91, respectively) and trauma-related distress (OR: 0.83, 95% CI: 0.73–0.94; OR: 0.86, 95% CI: 0.75–0.98, respectively). Compared to having a secure attachment style, having a preoccupied or a fearful attachment was associated with higher odds of developing psychological distress symptomatology (OR: 7.76, 95% CI: 2.57–24.86; OR: 2.55, 95% CI: 1.12–5.89, respectively). High levels of emotional stability were significantly and negatively associated with symptoms of both psychological distress (OR: 0.65, 95% CI: 0.53–0.80) and trauma-related distress (OR: 0.76, 95% CI: 0.59–0.96) under the cutoff. Furthermore, scoring higher on the extroversion dimension was significantly and negatively associated with symptoms of psychological distress (OR: 0.81, 95% CI: 0.67–0.96), while scoring higher on the openness dimension was associated with symptoms of trauma-related distress above the cutoff (OR: 1.29, 95% CI: 1.02–1.65). Lastly, compared to physicians, psychologists showed a significant reduction in psychological distress (OR: 0.29, 95% CI: 0.09–0.88) as well as trauma-related distress (OR: 0.17, 95% CI: 0.00–0.44).

### 3.4. Adjusted Vulnerability and Protective Factors

In the multivariable-adjusted regression model (Table 4), scoring higher on the emotional stability dimension of personality was associated with lower symptoms of general psychological distress (aOR: 0.58, 95% CI: 0.41–0.79) and pandemic-related distress (aOR: 0.62, 95% CI: 0.41–0.90). Additionally, having higher scores in the ability to understand others’ emotional states (CRE) was associated with lower odds of developing psychological distress (aOR: 0.78, 95% CI: 0.63–0.93). Lastly, when comparing fearful attachment to secure attachment (reference category), the aOR for psychological distress was 4.73 (95% CI: 1.45–17.04).

## 4. Discussion

This is one of few studies to date that have investigated the relationship between personal factors (specifically, personality domains, attachment style, and metacognitive functioning) and the pandemic’s impact on HCWs’ psychological distress.

We found that about one-fourth of HCWs had clinically relevant general psychological distress, while just over one in ten presented with clinically relevant pandemic-related distress. The estimated self-reported rate of general psychological distress (26.5%) among our sample participants (surveyed over the period June–October 2020) was slightly lower than the rate reported by other Italian studies that used subscales measuring the same constructs to assess the general Italian population [36,55,56] and healthcare workers [55,57,58] from March to May 2020. This difference could be due to the fact that our data were collected after the end of the first COVID-19 pandemic, when restrictions on movement and social life were restored. However, the reported rates are notably higher than those found in pre-pandemic Italian population-based studies [59]. Our findings show that 13.8% of HCWs suffer from pandemic-related psychological distress, a percentage slightly lower than that reported by other Italian COVID-19 studies that used the same measurements over the first trimester of the pandemic outbreak in Italy [43,56], when stringent disease containment measures were in place.

These data suggest that the COVID-19 pandemic has directly (pandemic-related distress) and indirectly (general distress) affected HCWs’ mental health and that its impact on the quality of their working life was still present, though at a lesser extent, three months after the start of the outbreak. One reason for this persistence may be the ongoing process of familiarization and adjustment of both healthcare systems and HCWs during this crisis. Our results overall indicate the importance of regular mental status monitoring and psychological support during the next phases of the COVID-19 pandemic and the related containment measures. This is particularly important considering that mental well-being is crucial for HCWs’ own general health and well-being as well as for their effectiveness and productivity at work, which impacts the quality and provision of medical care and patients’ safety and outcomes.

The main aim of this study is to identify personal factors relating to psychological distress to identify potential targets for screening and identifying HCWs in need of additional support during the COVID-19 pandemic. Our results indicate that the personality dimension of emotional stability (i.e., the tendency to stay calm in stressful situations) was one such factor. It was the only protective factor for pandemic-related distress and one of two protective factors for general psychological distress. This result aligns with evidence from previous pandemic studies on the general population [36,60,61] as well as with results from a recent metasynthesis, indicating that emotional stability is associated with better mental health [62]. Moreover, it should be noted that neuroticism is typically associated with reactivity to stressors [63], making it likely that this specific personality dimension affects how people perceive and evaluate the pandemic.

Additionally, general psychological distress was associated with two person-level variables: the ability to understand others’ emotional states (protective factor) and a fearful attachment style (risk factor). These findings align with previous literature on psychological distress, attachment styles, and metacognition. Indeed, the ability to recognize emotions and, more generally, metacognitive capacities has been shown to mitigate traumatic stress and, thus, positively influence quality of life [64,65,66], while a fearful–avoidant attachment style is usually associated with poorer mental health outcomes [67,68,69].

This study deepens our understanding of how the pandemic has influenced Italian healthcare facilities and workers and may be crucial in guiding the development and implementation of effective crisis responses and, more broadly, supporting and strengthening perinatal health systems. From this perspective, crises are also times of opportunity [7]. The COVID-19 pandemic has caused us to rethink how to improve access to and implementation of perinatal healthcare services. The improvements forced by the current pandemic will be useful during the next phases as well as future possible national or global health crises.

Overall, our results stress the importance of considering person-level variables in assessing the risk and protective factors of crisis workers and demonstrate effects independent of and beyond basic demographic and occupational variables. These findings indicate that HCWs as individuals are not equally impacted by the pandemic and its related measures and, thus, highlight the importance of considering intra-personal features during the COVID-19 pandemic or other stressful situations. Future research should investigate how to efficiently identify HCWs with personal risk factors for psychological distress, improve these specific emotional and psychological characteristics, and psychologically support HCWs during periods of crisis and stress.

### Strengths and Limitations

The strengths of this study include our focus on the healthcare worker population as well as on person-level variables, the enrollment of HCWs representing different professional roles, and the use of validated questionnaires. However, some limitations exist. First, the cross-sectional design did not enable us to identify and analyze symptom trajectories. Second, a non-probability and snowball sampling method was utilized instead of a random sampling method; thus, our sample may not be representative of the typical population of HCWs. Third, an online survey was conducted to access HCWs due to the ongoing pandemic. Online studies are a data collection method that may lead to non-response bias that, in turn, can further limit the generalizability of the results. Fourth, not all questionnaires were completely filled in, which may suggest that the survey was too long, and some participants gave up before they were able to complete it. Fifth, the questionnaires used in the survey were not randomly administered, which explains the difference between the number of participants who filled in the different questionnaires. Sixth, we had to merge HCWs other than physicians, midwives, and psychologists under “other’ healthcare professionals” to obtain statistical power, despite that some professional roles (such as nurses) are likely to be more vulnerable to the pandemic emergency than, for example, speech therapists. It must be noted that this sample included an unusually small number of physicians and nurses, groups that, in reality, constitute the largest professional groups among the medical professions. Seventh, we assumed that all participants were psychologically healthy. Lastly, structured or semi-structured interviews were not used for the assessment of both psychological distress and personal features.

## 5. Conclusions

The current study found high rates of self-reported general psychological distress in a sample of Italian HCWs who were active between the first and the second wave of the COVID-19 pandemic in Italy. The main finding is that HCWs’ person-level factors (personality traits, attachment styles, and metacognition) may serve as a protective factor against psychological distress. Considering our findings and the potentially harmful effects of psychological distress on HCWs’ quality of life and professional performance, it is of primary importance to conduct a baseline assessment of personal factors (i.e., personality traits, attachment style, and ability to understand emotional states) and provide timely and regular screenings of psychological distress in order to reduce HCWs’ burden of mental health concerns and to improve their performance at work and, thus, healthcare provisions overall.

## Figures and Tables

**Table 1:** Socio-demographic, occupational-related, and person-level characteristics of HCWs.

Characteristic	Study Sample *n* = 235
Socio-Demographic and Professional-Related Data Age	
Mean ± SD	44.40 ± 11.46
Range	26–66
Gender	
Female	219 (93.2%)
Educational level	
Secondary	13 (6.0%)
University	107 (49.5%)
Post-university	96 (44.5%)
Work position	
Freelancer or temporary employment contract	60 (25.5%)
Permanent employment	175 (74.5%)
Professional role	
Physician	34 (14.5%)
“Other” position	72 (30.6%)
Midwifery	71 (30.2%)
Psychologist	58 (24.7%)
Workplace	
Community-based	147 (69.4%)
Hospital-based	60 (30.6%)
Work experience (years)	
≤5	71 (30.2%)
6–15	72 (30.6%)
≥16	92 (39.1%)
Working during pandemic	
As usual	94 (40.3%)
More than usual	68 (29.2%)
Less than usual	71 (30.5%)
Working in a COVID-19 unit	
Yes	13 (6.5%)
No	222 (94.5%)
Person-level features	
Attachment style	
Secure	90 (48.9%)
Dismissing	26 (14.1%)
Preoccupied	17 (9.2%)
Fearful	51 (27.7%)
Metacognitive functioning (mean ± SD)	
CRE	12.73 ± 3.45
CRC	18.23 ± 3.53
CDD	33.24 ± 4.86
CDP	12.62 ± 2.40
BFI personality traits (mean ± SD)	
Agreeableness	7.23 (1.54)
Conscientiousness	8.23 (1.40)
Emotional stability	6.85 (1.40)
Extraversion	6.20 (1.80)
Openness	6.65 (1.83)

Note: CDD = ability to judge the distance between objects and from an object to oneself; CDP = ability to ponder situations and problems; CRC = ability to understand causal relationships; CRE = ability to understand others’ emotional states. A minority of participants did not complete the measures for educational level (*n* = 11), workplace (*n* = 22), working during pandemic (*n* = 2), attachment style (*n* = 8), BFI: Big Five Inventory personality traits (*n* = 2), and metacognitive functioning (*n* = 12).

**Table 2:** Statistically significant socio-demographic, occupational-related, and person-level differences among HCWs and the prevalence of general and pandemic-related psychological distress.

Characteristic	General Psychological Distress			Pandemic-Related Psychological Distress		
	No	Yes	*p*	No	Yes	*p*
Work position			0.841			**0.038**
Freelancer or temporary employee	34 (23.6%)	13 (25.0%)		52 (26.9%)	3 (9.7%)	
Permanent employment	110 (76.4%)	39 (75.0%)		141 (73.1%)	28 (90.3%)	
Professional role			0.071			**0.008**
Physician	20 (13.9%)	10 (19.2%)		27 (14.0%)	7 (22.6%)	
“Other” position	42 (29.2%)	16 (30.8%)		60 (31.1%)	8 (25.8%)	
Midwifery	40 (27.8%)	20 (38.5%)		53 (27.5%)	15 (48.4%)	
Psychologist	42 (29.2%)	6 (11.5%)		53 (27.5%)	1 (3.2%)	
Working during pandemic			0.393			**0.049**
Missing data	0	1		1	1	
As usual	62 (43.1%)	19 (37.3%)		76 (39.6%)	12 (40.0%)	
More than usual	39 (27.1%)	19 (37.3%)		54 (28.1%)	14 (46.7%)	
Less than usual	43 (29.9%)	13 (25.5%)		62 (32.3%)	4 (13.3%)	
Attachment style			**0.001**			0.241
Missing data	8	4		35	5	
Secure	76 (55.9%)	14 (29.2%)		80 (50.6%)	10 (38.5%)	
Dismissing	19 (14.0%)	7 (14.6%)		23 (14.6%)	3 (11.5%)	
Preoccupied	7 (5.1%)	10 (20.8%)		12 (7.6%)	5 (19.2%)	
Fearful	34 (25.0%)	17 (35.4%)		43 (27.2%)	8 (30.8%)	
Metacognitive functioning CRE			**<0.001**			**0.005**
Missing data	12	6		37	8	
Mean (SD)	13.41 (2.93)	10.52 (3.59)		13.00 (3.35)	10.87 (3.60)	
Metacognitive functioning CRC			**<0.001**			**0.034**
Missing data	12	6		37	8	
Mean (SD)	18.76 (3.08)	16.41 (3.640)		18.45 (3.50)	16.78 (3.42)	
BFI Emotional stability			**<0.001**			**0.019**
Missing data	2	1		26	4	
Mean (SD)	7.17 (1.65)	5.96 (1.77)		6.96 (1.73)	6.11 (1.76)	
BFI Extraversion			**0.013**			0.693
Missing data	2	1		26	4	
Mean (SD)	6.39 (1.70)	5.67 (1.97)		6.22 (1.81)	6.07 (1.75)	
BFI Openness			0.144			**0.037**
Missing data	2	1		26	4	
Mean (SD)	6.54 (1.87)	6.98 (1.69)		6.54 (1.78)	7.33 (2.02)	

Note: CRC = ability to understand causal relationships; CRE = ability to understand others’ emotional states. BFI = Big Five Inventory. Bold *p*-values indicate statistical significance.

**Table 3:** Associations between socio-demographic, occupational-related, and person-level variables among HCWs and (general and pandemic-related) psychological distress.

Predictors	General Psychological Distress		Pandemic-Related Psychological Distress	
	OR (95% CI)	*p*	OR (95% CI)	*p*
Age (per 1 year increase)	0.97 (0.94–1.00)	0.063	1.00 (0.97–1.04)	0.774
Gender (ref. Male)				
Female	1.32 (0.39–6.03)	0.677	1.05 (0.27–6.96)	0.948
Work position (ref. Permanent employment)				
Freelancer or temporary employment contract	1.11 (0.52–2.28)	0.788	0.29 (0.07–0.86)	**0.048**
Professional role (ref. Physician)				
Midwifery	1.00 (0.40–2.59)	1.000	1.09 (0.41–3.15)	0.865
“Other” position	0.71 (0.27–1.91)	0.493	0.52 (0.17–1.63)	0.253
Psychologist	0.29 (0.09–0.88)	**0.032**	0.07 (0.00–0.44)	**0.017**
Workplace (ref. hospital-based)				
Community-based	0.87 (0.42–1.87)	0.708	0.55 (0.23–1.30)	0.162
Work experience (ref. ≤5 years)				
6–15	0.88 (0.39–1.98)	0.751	0.80 (0.27–2.38)	0.692
≥16	0.71 (0.32–1.57)	0.398	1.56 (0.64–4.09)	0.341
Work position (ref. As usual)				
Less than usual	1.04 (0.46–2.34)	0.922	0.40 (0.11–1.22)	0.132
More than usual	1.68 (0.79–3.61)	0.181	1.62 (0.69–3.83)	0.264
Working in a COVID-19 unit (ref. No)				
Yes	0.61 (0.09–2.48)	0.539	1.13 (0.17–4.51)	0.874
Attachment style (ref. Secure)				
Dismissing	2.00 (0.68–5.55)	0.942	1.04 (0.22–3.75)	0.951
Fearful	2.55 (1.12–5.89)	**0.026**	1.52 (0.54–4.15)	0.410
Preoccupied	7.76 (2.57–24.86)	**<0.001**	3.33 (0.91–11.24)	0.056
BFI personality traits				
Agreeableness	0.87 (0.70–1.07)	0.185	1.10 (0.84–1.45)	0.501
Conscientiousness	0.81 (0.64–1.02)	0.074	0.89 (0.67–1.19)	0.432
Emotional stability	0.65 (0.53–0.80)	**<0.001**	0.76 (0.59–0.96)	**0.033**
Extroversion	0.81 (0.67–0.97)	**0.023**	0.95 (0.75–1.19)	0.652
Openness	1.13 (0.94–1.36)	0.187	1.29 (1.02–1.65)	**0.036**
Metacognitive functioning				
CDD	0.94 (0.87–1.01)	0.104	1.00 (0.99–1.55)	0.085
CDP	1.11 (0.95–1.32)	0.218	1.22 (0.79–1.65)	0.504
CRC	0.81 (0.73–0.91)	**<0.001**	0.86 (0.75–0.98)	**0.025**
CRE	0.77 (0.68–0.86)	**<0.001**	0.83 (0.73–0.94)	**0.005**

Note: OR = odds ratio. Bold *p*-values indicate statistical significance. BFI = Big Five Inventory; CDD = ability to judge the distance between objects and from an object to oneself; CDP = ability to ponder situations and problems; CRC = ability to understand causal relationships; CRE = ability to understand others’ emotional states.

**Table 4:** Adjusted odds ratios and confidence intervals of the associations with general and pandemic-related psychological distress.

Predictors	General Psychological Distress		Pandemic-Related Psychological Distress	
	aOR (95% CI)	*p*	aOR (95% CI)	*p*
Age (per 1 year increase)	0.99 (0.94–1.03)	0.563	1.02 (0.96–1.08)	0.594
Gender (ref. Male)				
Female	2.82 (0.49–22.28)	0.279	0.85 (0.15–6.99)	0.868
Professional role (ref. Physician)				
Midwifery	0.71 (0.16–3.15)	0.651	1.75 (0.35–10.14)	0.508
“Other” position	0.94 (0.24–3.93)	0.936	1.11 (0.22–6.27)	0.905
Psychologist	0.25 (0.04–1.58)	0.147	0.00 (0.00–NA)	0.991
Work position (ref. As usual)				
Less than usual	1.45 (0.43–4.96)	0.546	1.53 (0.26–8.15)	0.623
More than usual	1.89 (0.61–6.10)	0.275	2.80 (0.74–11.44)	0.137
Working position (ref. Permanent employment)				
Freelancer or temporary employment contract	2.78 (0.82–10.10)	0.107	1.30 (0.20–7.36)	0.773
Attachment style (ref. Secure)				
Dismissing	1.05 (0.26–4.22)	0.942	1.19 (0.16–7.63)	0.856
Fearful	4.73 (1.45–17.04)	**0.013**	2.37 (0.57–10.63)	0.241
Preoccupied	4.53 (0.87–23.52)	0.077	0.86 (0.11–5.95)	0.883
BFI personality traits				
Agreeableness	1.14 (0.81–1.62)	0.474	1.50 (0.94–2.54)	0.104
Conscientiousness	0.83 (0.57–1.19)	0.308	0.78 (0.50–1.21)	0.274
Emotional stability	0.58 (0.41–0.79)	**0.001**	0.62 (0.41–0.90)	**0.016**
Extroversion	0.94 (0.72–1.21)	0.614	1.03 (0.76–1.39)	0.854
Openness	1.30 (0.98–1.76)	0.082	1.36 (0.98–1.95)	0.081
Metacognitive functioning				
CDD	1.06 (0.93–1.21)	0.386	1.10 (0.94–1.30)	0.231
CDP	1.21 (0.91–1.62)	0.198	1.13 (0.79–1.65)	0.504
CRC	0.92 (0.77–1.10)	0.361	0.95 (0.75–1.19)	0.642
CRE	0.78 (0.63–0.30)	**0.010**	0.83 (0.66–1.02)	0.088
Observations	176		176	
R^2^ Tjur	0.352		0.245	

Note: aOR = adjusted odds ratio. Bold *p*-values indicate statistical significance. BFI = Big Five Inventory; CDD = ability to judge the distance between objects and from an object to oneself; CDP = ability to ponder situations and problems; CRC = ability to understand causal relationships; CRE = ability to understand others’ emotional states.

## Data Availability

The complete dataset is available from the corresponding author upon request.

## Supplementary Materials

The following are available online at https://www.mdpi.com/article/10.3390/ijerph182211843/s1, Table S1: Demographic, occupational, and person-level characteristics of HCWs, and prevalence of general and pandemic-related psychological distress.
