# Supplementary material for: Psychological Distress in Healthcare Workers between the First and Second COVID-19 Waves: The Role of Personality Traits, Attachment Style, and Metacognitive Functioning as Protective and Vulnerability Factors
Source: Int J Environ Res Public Health. 2021 Nov 11;18(22):11843. doi: 10.3390/ijerph182211843 (PMC8623543; doi:10.3390/ijerph182211843)
Supplement: Supplementary file 1 [file ijerph-18-11843-s001.zip › ijerph-1399187-supplementary.pdf]

Table S1. Demographic, occupational, and person-level characteristics of HCWs, and prevalence of general and pandemic-related psychological distress.

Characteristic	General psychological distress			Pandemic-related psychologicl distress		
	No	Yes	*p.*	No	Yes	*p.*
Age			0.086			0.800
Mean (SD)	45.57 (10.98)	42.44 (11.86)		44.50 (11.65)	45.06 (10.89)	
Range	26–66	24–66		24–66	26–60	
Gender			0.654			0.953
Male	11 (7.6%)	3 (5.8%)		13 (6.7%)	2 (6.5%)	
Female	133 (92.4%)	49 (94.2%)		180 (93.3%)	29 (93.5%)	
Educational level			0.532			0.345
Missing data	11	4		18	0	
Secondary	8 (6.0%)	1 (2.1%)		10 (5.7%)	2 (6.5%)	
University	63 (47.4%)	25 (52.1%)		84 (48.0%)	19 (61.3%)	
Post-University	62 (46.6%)	22 (45.8%)		81 (46.3%)	10 (32.3%)	
Working position			0.841			**0.038**
Freelancer or temporary employee	34 (23.6%)	13 (25.0%)		52 (26.9%)	3 (9.7%)	
Permanent employment	110 (76.4%)	39 (75.0%)		141 (73.1%)	28 (90.3%)	
Professional role			0.071			**0.008**
Physician	20 (13.9%)	10 (19.2%)		27 (14.0%)	7 (22.6%)	
"Other" position	42 (29.2%)	16 (30.8%)		60 (31.1%)	8 (25.8%)	
Midwifery	40 (27.8%)	20 (38.5%)		53 (27.5%)	15 (48.4%)	
Psychologist	42 (29.2%)	6 (11.5%)		53 (27.5%)	1 (3.2%)	
Workplace			0.572			0.173
Missing data	22	8		31	5	
Community-based	86 (70.5%)	29 (65.9%)		115 (71.0%)	15 (57.7%)	
Hospital-based	36 (29.5%)	15 (34.1%)		47 (29.0%)	11 (42.3%)	
Work experience (years)			0.770			0.352
≤5	38 (26.4%)	16 (30.8%)		57 (29.5%)	8 (25.8%)	
6-15	46 (31.9%)	17 (32.7%)		62 (32.1%)	7 (22.6%)	
≥16	60 (41.7%)	19 (36.5%)		74 (38.3%)	16 (51.6%)	
Working during pandemic			0.393			**0.049**
Missing data	0	1		1	1	
As usual	62 (43.1%)	19 (37.3%)		76 (39.6%)	12 (40.0%)	
More than usual	39 (27.1%)	19 (37.3%)		54 (28.1%)	14 (46.7%)	
Less than usual	43 (29.9%)	13 (25.5%)		62 (32.3%)	4 (13.3%)	
Working in a COVID-19 unit			0.519			0.868
Yes	9 (6.2%)	2 (3.8%)		11 (5.7%)	2 (6.5%)	
No	135 (93.8%)	50 (96.2%)		182 (94.3%)	29 (93.5%)	
Attachment style			**0.001**			0.241
Missing data	8	4		35	5	
Secure	76 (55.9%)	14 (29.2%)		80 (50.6%)	10 (38.5%)	
Dismissing	19 (14.0%)	7 (14.6%)		23 (14.6%)	3 (11.5%)	
Preoccupied	7 (5.1%)	10 (20.8%)		12 (7.6%)	5 (19.2%)	
Fearful	34 (25.0%)	17 (35.4%)		43 (27.2%)	8 (30.8%)	
Metacognitive functioning CRE			**< 0.001**			**0.005**
Missing data	12	6		37	8	
Mean (SD)	13.41 (2.93)	10.52 (3.59)		13.00 (3.35)	10.87 (3.60)	
Metacognitive functioning CRC			**< 0.001**			**0.034**
Missing data	12	6		37	8	
Mean (SD)	18.76 (3.08)	16.41 (3.64)		18.45 (3.50)	16.78 (3.42)	
Metacognitive functioning CDD			0.070			0.946
Missing data	12	6		37	8	
Mean (SD)	33.70 (4.62)	32.22 (5.05)		33.23 (4.81)	33.30 (5.31)	
Metacognitive functioning CDP			0.159			0.099
Missing data	12	6		37	8	

Mean (SD)	12.55 (2.32)	13.09 (1.84)		12.51 (2.45)	13.39 (1.95)	
BFI Agreeableness			0.208			0.512
Missing data	2	1		26	4	
Mean (SD)	7.32 (1.48)	7.00 (1.68)		7.20 (1.55)	7.41 (1.45)	
BFI Conscientiousness			0.058			0.449
Missing data	2	1		26	4	
Mean (SD)	8.35 (1.33)	7.92 (1.52)		8.26 (1.41)	8.04 (1.34)	
BFI Emotional stability			**< 0.001**			**0.019**
Missing data	2	1		26	4	
Mean (SD)	7.17 (1.65)	5.96 (1.77)		6.96 (1.73)	6.11 (1.76)	
BFI Extraversion			**0.013**			0.693
Missing data	2	1		26	4	
Mean (SD)	6.39 (1.70)	5.67 (1.97)		6.22 (1.81)	6.07 (1.75)	
BFI Openness			0.144			**0.037**
Missing data	2	1		26	4	
Mean (SD)	6.54 (1.87)	6.98 (1.69)		6.54 (1.78)	7.33 (2.02)	

Note: CDD = ability to judge the distance of objects from one another and from ourselves; CDP = ability to ponder situations and problems; CRC = ability to understand causal relationships; CRE = ability to understand others' emotional states. Bold p-values indicate statistical significance.
